# Polymorphism in HIV-1 dependency factor PDE8A affects gene expression and HIV-1 replication in primary macrophages

**DOI:** 10.1186/1742-4690-8-S2-O37

**Published:** 2011-10-03

**Authors:** Thijs Booiman, Sebastiaan Bol, Evelien Bunnik, Perry Moerland, Karel van Dort, Jerome Strauss, Margit Síeberer, Hanneke Schuitemaker, Neeltje Kootstra, Angélique van't Wout

**Affiliations:** 1 Department of Experimental Immunology, Academic Medical Center of the University of Amsterdam, Amsterdam, 1105AZ, The Netherlands; 2 Department Bioinformatics, Academic Medical Center of the University of Amsterdam, Amsterdam, 1 105AZ, The Netherlands; 3 Department of Obstetrics and Gynecology, Virginia Commonwealth University, School of Medicine, Richmond, VA, 23298, USA

## Background

The limited size of the human immunodeficiency virus 1 (HIV-1) genome and the small number of proteins it encodes make the virus highly dependent on host proteins for its replication. Four genome-wide RNAi screens have recently identified a large number of HIV-1 dependency factors (HDFs), with the majority of these proteins never before associated with HIV-1 replication. Recently, we reported more than 3 log variation in the ability of HIV-1 to replicate in monocyte derived macrophages (MDM) derived from >4OO healthy seronegative blood donors. In our present study we determined whether single nucleotide polymorphisms (SNPs) in the genes encoding the newly identified HDFs were associated with this variation in HIV-1 replication.

## Materials and methods

DNA from Caucasian donors whose MDM had low (n=96) or high (n=96) viral Gag p24 production, was used for genome-wide SNP genotyping. Linear regression assuming an additive model was used to test for association between the genotype of HDF SNPs and HIV-1 replication in MDM.

## Results

We found a significant association between the minor allele of SNP rs2304418 located in the phosphodiesterase 8a (*PDE8A*) gene and lower HIV-1 replication (*p*=2.4×10^-6^), even after correction for multiple testing. This finding was independent of the *CCR5* Δ32 genotype. The minor allele of SNP rs2304418 was also significantly associated with lower *PDE8A* mRNA levels in MDM (*p*=8.3×10^-5^) and *PDE8A* mRNA levels correlated with HIV-1 replication. Resequencingof the promotor and untranslated regions of the *PDE8A* mRNA did not reveal novel SNPs likely to be the causative variant.

## Conclusions

Our finding is in agreement with the reported finding that RNAi knock-down of *PDE8A* resulted in lower HIV-1 replication. *PDE8A* is highly expressed in macrophages and specifically catalyzes the hydrolysis of cAMP to AMP. We are currently investigating at which level of the virus life cycle *PDE8A* affects HIV-1 replication.

